# Heart-brain synchronization breakdown in Parkinson’s disease

**DOI:** 10.1038/s41531-022-00323-w

**Published:** 2022-05-30

**Authors:** Martin Iniguez, Antonio Jimenez-Marin, Asier Erramuzpe, Marian Acera, Beatriz Tijero, Ane Murueta-Goyena, Rocio Del Pino, Tamara Fernandez, Mar Carmona‑Abellan, Alberto Cabrera-Zubizarreta, Juan Carlos Gómez‑Esteban, Jesus M. Cortes, Inigo Gabilondo

**Affiliations:** 1 School of Engineering, University Carlos III of Madrid, Madrid, Spain; 2 Bernstein Center for Computational Neuroscience, Berlin, Germany; 3 Computational Neuroimaging Group, Biocruces Bizkaia Health Research Institute, Barakaldo, Spain; 4 Biomedical Research Doctorate Program, University of the Basque Country (UPV/EHU), Leioa, Spain; 5 Mechanics and Industrial Production, Mondragon Unibertsitatea, Higher Polytechnic School, Mondragon, Gipuzkoa, Spain; 6 Neurodegenerative Diseases Group, Biocruces Bizkaia Health Research Institute, Barakaldo, Bizkaia Spain; 7 Department of Neurosciences, University of the Basque Country (UPV/EHU), Leioa, Spain; 8 Osatek Magnetic Resonance Imaging Unit, Galdakao Hospital, Galdakao, Spain; 9 Department of Cell Biology and Histology, University of the Basque Country (UPV/EHU), Leioa, Spain; 10 Ikerbasque: The Basque Foundation for Science, Bilbao, Spain

**Keywords:** Parkinson's disease, Parkinson's disease

## Abstract

Heart rate variability (HRV) abnormalities are potential early biomarkers in Parkinson’s disease (PD) but their relationship with central autonomic network (CAN) activity is not fully understood. We analyzed the synchronization between HRV and brain activity in 31 PD patients and 21 age-matched healthy controls using blood-oxygen-level-dependent (BOLD) signals from resting-state functional brain MRI and HRV metrics from finger plethysmography recorded for 7.40 min. We additionally quantified autonomic symptoms (SCOPA-AUT) and objective autonomic cardiovascular parameters (blood pressure and heart rate) during deep breathing, Valsalva, and head-up tilt, which were used to classify the clinical severity of dysautonomia. We evaluated HRV and BOLD signals synchronization (HRV-BOLD-sync) with Pearson lagged cross-correlations and Fisher’s statistics for combining window-length-dependent HRV-BOLD-Sync Maps and assessed their association with clinical dysautonomia. HRV-BOLD-sync was lower significantly in PD than in controls in various brain regions within CAN or in networks involved in autonomic modulation. Moreover, heart-brain synchronization index (HBSI), which quantifies heart-brain synchronization at a single-subject level, showed an inverse exposure–response relationship with dysautonomia severity, finding the lowest HBSI in patients with severe dysautonomia, followed by moderate, mild, and, lastly, controls. Importantly, HBSI was associated in PD, but not in controls, with Valsalva pressure recovery time (sympathetic), deep breathing E/I ratio (cardiovagal), and SCOPA-AUT. Our findings support the existence of heart-brain de-synchronization in PD with an impact on clinically relevant autonomic outcomes.

## Introduction

Autonomic manifestations are frequent in Parkinson’s disease (PD)^1,2^, have been associated with disease progression and shortened survival^3^, and are considered prognostic biomarkers and key features of prodromal PD^4^. Besides orthostatic hypotension, non-invasive hemodynamic tests demonstrate that PD patients have other well-defined cardiovascular autonomic abnormalities such as alterations in heart rate variability (HRV)^5–10^. HRV is a widely used marker of autonomic activity and an indicator for heart’s neural control at the sinus node level^11^. Time and frequency-domain indices of HRV can be obtained by several metrics, using linear or non-linear mathematical tools, that are associated with HRV control of sympathetic and parasympathetic autonomic nervous systems^12^. HRV abnormalities in PD are common but often unrecognized, although they are gaining attention as potential biomarkers in PD^7,13^. Alterations in HRV have been detected in premotor phases of PD^5,8^, being associated with increased risk of PD^14^ and with the development of cognitive disability in PD^15^. The physiological regulation of HRV (and its derived metrics) are influenced by several factors, such as mood, stress, and alertness, afferent inputs from baroreceptors and chemoreceptors, and the effects of cardiovascular, endocrine, and respiratory systems, that ultimately modulate the balance between sympathetic and parasympathetic (cardiovagal) efferent branches of the autonomic nervous system (ANS)^16^. The observations in humans and experimental studies in animals have helped to identify the central autonomic network (CAN), which encompasses the main integrative control centers of the ANS^17–19^. The use of non-invasive brain-imaging methods, such as functional MRI (fMRI), has also helped to characterize CAN in healthy subjects^20^, and its modulatory role for autonomic parameters such as HRV^21–25^. In PD, neuropathological evidence supports the involvement of structures belonging to CAN^26^, while the degree of sympathetic myocardial denervation does not fully explain the observed heart rate abnormalities^27^. Simultaneous acquisition of resting fMRI and photoplethysmography (PPG) in PD patients has shown a correlation between regional blood oxygenation level-dependent (BOLD) signal and the dynamics of specific cardiovagal HRV parameters, and this occurred at several structures belonging to CAN and other regions for central autonomic control^23,24^. In the present study, we extend these results by assessing heart-brain synchronization in a broad set of HRV metrics reflecting both sympathetic and cardiovagal activity, pointing out the differentiated role played by CAN, and quantifying their association with relevant autonomic outcomes in PD.

## Results

### Demographics, neurological, and autonomic features of participants

The demographics and clinical characteristics of study participants are detailed in Table 1. Briefly, patients had a mean age of 54.6 years, 57% were male and had 12.9 years of education, with no significant differences compared to controls. General cognitive performance, measured with Montreal Cognitive Assessment (MoCA), was lower in patients as compared to controls, but the difference was not significant. In the subgroup of patients and controls that additionally underwent an assessment of autonomic manifestations, evaluated with Scales for Outcomes in Parkinson’s Disease-Autonomic questionnaire (SCOPA-AUT) and cardiovascular autonomic tests (See Methods and Supplementary Table S1), SCOPA-AUT total score was significantly higher (*p* < 0.05) (meaning more severe dysautonomia symptoms) in patients, being twice higher than in controls. Similarly, patients had objective abnormalities in cardiovascular autonomic tests compared to controls. First, the prevalence of orthostatic hypotension (OH) was significantly higher in patients (70%) than in controls (15.4%) (*p* = 0.017). Second, blood pressure recovery time (PRT) and the change of systolic blood pressure from baseline to phase IV (ΔSBP phase IV) of Valsalva maneuver, represent, respectively, the early and delayed effects on blood pressure of the sympathetic system during Valsalva, were the cardiovascular parameters showing the most significant group differences (*p* = 0.021 and *p* = 0.002, respectively). Lastly, the deep breathing expiratory-to-inspiratory ratio (E/I ratio), one of the main cardiovagal parameters, was lower in patients, but such a difference was at the limit of significance (*p* = 0.051). All these results show that, compared to healthy controls, PD patients had significant autonomic manifestations and objective cardiovascular autonomic abnormalities.

**Table 1:** Demographics and neurological characteristics of participants.

	Controls	iPD	*p*
*n*	21	31	NA
Age, years	54.6 (2.7)	59.0 (1.5)	0.067
Females (%)	9 (43)	11 (35)	0.592
Education, years	12.9 (1.1)	10.2 (0.7)	0.255
Disease duration, years	NA	5.6 (0.6)	NA
Age disease onset, years	NA	53.6 (1.4)	NA
UPDRS III score	NA	27.3 (2.0)	NA
HY score, median (IQR)	NA	2.0 (1.0–4.0)	NA
MoCA score	26.8 (0.7)	24.3 (0.6)	0.352
LEDD, mg/day	NA	594.6 (74.5)	NA

*p*-values were obtained with Student's *t*-test except for “Females” variable, for which Pearson's chi-squared test was used. Means and standard deviations are shown for participant columns unless specified in the table.

*iPD* idiopathic Parkinson’s disease, *UPDRS III* motor part of Unified Parkinson’s Disease Rating Scale, *HY score* Hoehn & Yahr score, *IQR* interquartile range, *MoCA* Montreal Cognitive Assessment, *LEDD* L-Dopa equivalent daily dose.

### Differences between patients and controls in HRV-BOLD Sync Maps

**Fig. 1:**
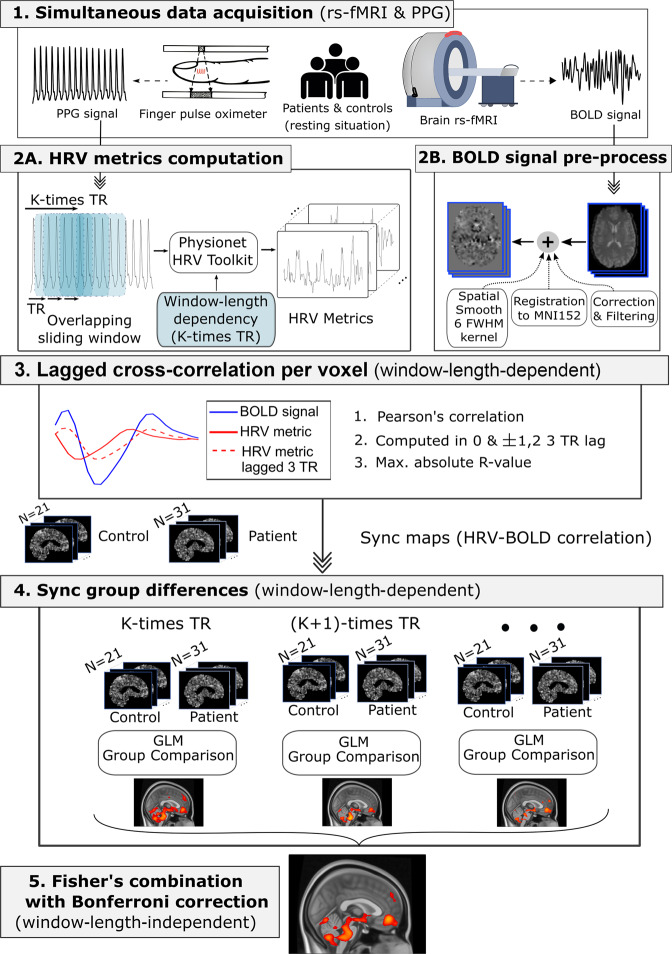
Methodological sketch. **1** Simultaneous and synchronous data acquisition for brain resting-state functional MRI (rs-fMRI) and finger photoplethysmography (PPG); **2A** Heart Rate Variability (HRV) metrics computation using the *PhysioNet Cardiovascular Signal Toolbox* and definition of *K* overlapping sliding time windows with integer multiples of the Repetition Time (TR) parameter of fMRI acquisition; **2B** Preprocessing of blood-oxygen-level-dependent (BOLD) signal from rs-fMRI encompassed slice-time correction, removal of movement and physiological noise, band-pass filtering, registration to the Montreal Neurological Institute 152 (MNI152) template and spatial smoothing with a 6 full width at half maximum (FWHM) kernel; **3** Maximum Lagged cross-correlations were assessed for building HRV-BOLD Sync Maps for each participant. One map was obtained per window length; **4** Group differences were assessed for the HRV-BOLD Sync Maps, separately using the maps obtained at different window lengths; **5** Group comparison results across different windows were combined using the Fisher’s statistic and Bonferroni-correction.

**Fig. 2:**
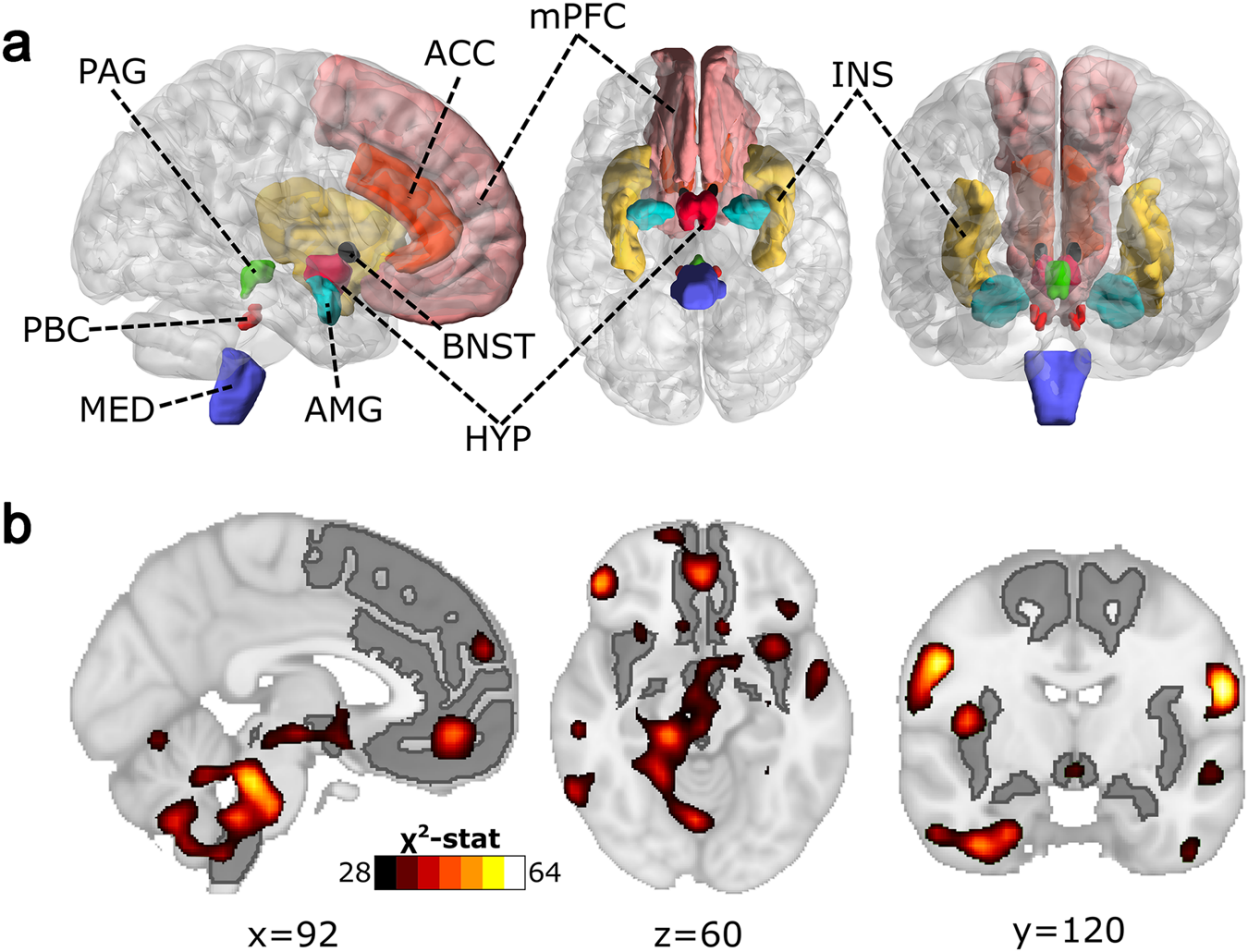
Central Autonomic Network and its intersection with HRV-BOLD de-synchronization maps. **a** Anatomical representation of the Central Autonomic Network (CAN) created by pooling several regions extracted from different public MRI atlases (see Methods section in main text). **b** Statistically significant brain regions after group comparisons with the contrast patient <control in *NNiqr*-BOLD synchronization maps and surviving multiple comparisons. CAN network is colored in gray. The map of significant results is shown in a color scale that represents the value of the X^2^ statistic of the Fisher’s test. Significant brain regions overlay with those belonging to CAN, including cerebellum, brainstem, lateral parietal-temporal cortex, medial prefrontal cortex, insula, hypothalamus, and anterior cingulate cortex (see Results section in main text for further details). HYP hypothalamus, INS insular cortex, ACC anterior cingulate cortex, AMG amygdala, mPFC medial prefrontal cortex, PAG periaqueductal gray matter, MED medulla, PBC parabrachial complex, BNST bed nucleus of the stria terminalis.

Following the methodological sketch shown in Fig. 1, no significant group differences were found in HRV-BOLD Sync Maps for the contrast patient > control, and therefore patients did not present any brain region with significant increased HRV-BOLD synchronization maps. However, group differences with the contrast control > patient were found for the following HRV parameters: (1) Time-domain metrics: The standard deviation of normal-to-normal (NN) heartbeat intervals (*SDNN*) (associated with sympathetic and cardiovagal modulation of HRV), the root-mean-square of differences between successive NN (*RMSSD*) (related to cardiovagal control of respiratory sinus arrhythmia) and the NN interquartile range (*NNiqr*) (accounting for Gaussian deviations in the peak-to-peak variability^28^); (2) Spectral-domain metrics: The power spectrum high-frequency component (*HF*) (associated with cardiovagal control of respiratory sinus arrhythmia); (3) Non-linear metrics: *SD*1 × *SD*2 which represents the balance between *SD1* (that accounts for baroreflex sensitivity and *HF*, thus, mainly cardiovagal activity) and *SD2* [that correlates with baroreflex sensitivity and low-frequency component of power spectrum (*LF*), related to both sympathetic and cardiovagal activity]^12,29,30^. Of note, it is important to highlight that control > patient differences for such HRV parameters occurred at specific window lengths (WL). Accordingly, *NNiqr* metric was the one with the highest across-window significance, with significant voxels surviving multiple comparisons correction in WL = 6, 7, 8, 9, and 10-Repetition Time (TR), having a maximum number of voxels in 7-TR (14,832 voxels) and a minimum in 10-TR (3,502 voxels). After Fisher’s combination of *p*-values of individual windows and Bonferroni-correction, *NNiqr* was the only metric providing significant regions, and thus, we chose it for the following analyses. Since in our analyses *NNiqr* was significantly correlated with sympathetic and/or cardiovagal HRV parameters (*HF*, *SD2*, *SD1xSD2*, and *RMSSD*) (See Methods and Supplementary Fig. S1), we assumed that *NNiqr* accounted for both cardiovagal and sympathetic activity. After group comparison in HRV-BOLD Sync Maps, the resulting *NNiqr-*derived maps accounting for the anatomical regions in which sync was smaller in patients as compared to controls and their correspondence with the CAN atlas (See Methods) are depicted in Fig. 2 and listed in Tables 2 and 3. Significant voxels were distributed bilaterally within cortical and subcortical structures of the encephalon in a relatively bilateral arrangement, highlighting in order of representation the cerebellum, brainstem, lateral parietal-temporal cortex (postcentral and supramarginal gyri), medial prefrontal cortex (mPFC) (medial-orbitofrontal gyrus), insula, hypothalamus, and anterior cingulate cortex. Interestingly, 30% of all significant voxels were in the cerebellum (“Cerebellum-cortex” label from Desikan atlas). More importantly, the *NNiqr*-BOLD sync-diff map showed a high overlap with most labels of the CAN atlas, with a large number of intersecting voxels located in mPFC, insula, and the upper third of the medulla (Fig. 2 and Table 3). Of note, the *NNiqr*-BOLD sync-diff map occupied 28% of the volume of the hypothalamus, 71% of the parabrachial complex in the pons, 56% of the superior third of the medulla, and 35% of the periaqueductal gray matter of the midbrain.

**Table 2:** Intersection between labels of Desikan atlas and difference map between patients and controls for *NNiqr*-BOLD synchronization.

Desikan atlas label	N voxels	% within label (bilateral)	% right hemisphere	% left hemisphere	Peak chi^2^	Peak x (MNI)	Peak y (MNI)	Peak z (MNI)
Cerebellum-cortex	42,455	29.68	15.64	14.04	75.97	−20	−56	−57
Brainstem	10,735	7.50	NA	NA	55.50	−3	−33	−30
Supramarginal	7840	5.48	3.21	2.27	49.11	63	−21	36
Postcentral	7486	5.23	2.98	2.25	57.62	−56	−6	20
Inferiortemporal	6885	4.81	3.55	1.26	54.75	51	−12	−33
Precentral	5105	3.57	2.42	1.15	61.78	−56	−5	21
Fusiform	4293	3.00	2.25	0.75	51.90	28	−11	−37
Medialorbitofrontal	3169	2.22	1.50	0.72	44.77	1	45	−9
Insula	2590	1.81	0.66	1.15	40.30	41	−7	8
Lateralorbitofrontal	2529	1.77	1.27	0.49	40.39	12	51	−21
Superiorparietal	2217	1.55	0.62	0.93	45.17	−12	−69	54
Lingual	1947	1.36	0.85	0.51	47.28	7	−70	−8
Middletemporal	1788	1.25	0.86	0.39	41.53	57	−27	−9
Ventraldc	1706	1.19	0.62	0.58	39.60	15	−27	−8
Inferiorparietal	1493	1.04	0.76	0.28	60.89	36	−66	36
Superior frontal	1473	1.03	0.77	0.26	38.47	−3	57	24
Superiortemporal	1343	0.94	0.12	0.82	37.17	−55	−47	21
Entorhinal	1320	0.92	0.69	0.23	58.20	27	−12	−33
Parahippocampal	1282	0.90	0.75	0.15	44.96	16	−31	−12
Rostralmiddlefrontal	1207	0.84	0.82	0.02	36.17	15	58	−17
Parsorbitalis	1185	0.83	0.76	0.07	48.83	47	40	−9
Parstriangularis	1132	0.79	0.79	<0.01	49.41	48	39	−9
Bankssts	1053	0.74	0.00	0.74	39.60	−51	−48	21
Parsopercularis	960	0.67	0.67	0.00	50.32	50	8	14
Temporalpole	670	0.47	0.32	0.15	39.29	29	8	−35
Rostralanteriorcingulate	473	0.33	<0.01	0.32	42.76	−1	42	−8
Caudalmiddlefrontal	416	0.29	0.00	0.29	32.85	−39	18	42
Hippocampus	382	0.27	0.25	0.02	43.09	16	−31	−8
Frontalpole	331	0.23	0.23	0.00	37.40	11	58	−17
Precuneus	315	0.22	0.12	0.10	38.18	−8	−68	53
Caudate	304	0.21	0.21	<0.01	34.43	9	15	−3
Accumbens	230	0.16	0.12	0.04	33.99	6	12	−3
Paracentral	228	0.16	0.16	<0.01	36.73	9	−39	69
Putamen	142	0.10	<0.01	0.10	32.81	−16	4	−14
Posteriorcingulate	51	0.04	0.04	0.00	30.36	9	3	42
Caudalanteriorcingulate	32	0.02	0.02	0.00	29.77	6	30	18
Lateraloccipital	19	0.01	0.01	0.00	30.02	27	−84	−19
Thalamus-proper	8	<0.01	<0.01	<0.01	29.70	3	−1	0
Isthmuscingulate	3	<0.01	0.00	<0.01	29.88	−18	−48	0
Corpuscallosum	2	<0.01	<0.01	0.00	29.42	5	16	−3
Amygdala	1	<0.01	<0.01	0.00	28.15	24	−5	−30

**Table 3:** Intersection between *NNiqr*-BOLD sync-difference map and CAN.

CAN atlas label	N voxels	% map within label	% label within map	Peak chi^2^	Peak x (MNI)	Peak y (MNI)	Peak z (MNI)
Medial Prefrontal Cortex	4973	3.48	3.98	44.77	1	45	−9
Insula	2590	1.81	7.06	40.30	41	−7	8
Medulla (superior third)	1477	1.03	56.42	44.89	11	−44	−47
Hypothalamus	836	0.58	28.33	36.45	0	−12	−9
Medulla (middle third)	749	0.52	28.61	49.00	−9	−45	−56
Anterior Cingulate Cortex	505	0.35	2.37	42.76	−1	42	−8
Medulla (inferior third)	219	0.15	8.37	49.79	−9	−45	−58
Periaqueductal gray matter	158	0.11	35.27	35.70	0	−27	−11
Parabranchial complex	114	0.08	71.25	53.33	−6	−33	−29
Bed nucleus stria terminalis	2	<0.01	0.40	28.55	4	3	0
Amygdala	1	<0.01	0.02	28.15	24	−5	−30

### Dysautonomia severity and heart-brain synchronization index

**Fig. 3:**
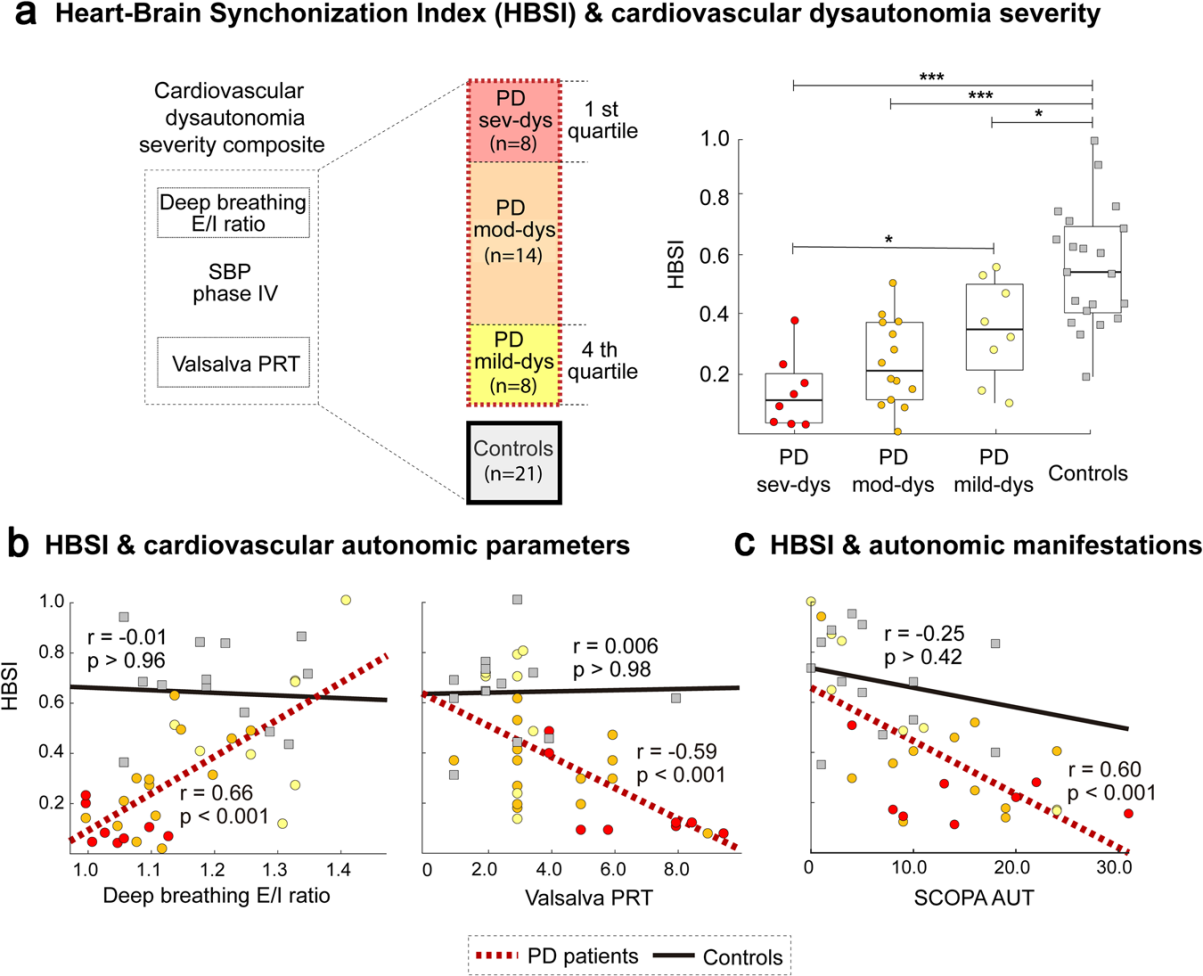
Relationship between heart-brain synchronization index and clinical autonomic outcomes. Panel **a** shows in its left side that the method to classify the severity of cardiovascular dysautonomia was based on a cardiovascular severity composite including E/I ratio, Valsalva PRT and ΔSBP phase IV (see Methods). PD patients with a composite value belonging to the 1st-quartile of the distribution of values were classified as PD sev-dys (*n* = 8), those belonging to the 4th-quartile as PD mild-dys (*n* = 8), and the rest of patients were considered as PD mod-dys (*n* = 14). On the right side, panel **a** shows a boxplot with differences in the distribution of heart-brain synchronization index (HBSI) (see Methods) for the dysautonomia severity PD subgroups and the controls (*n* = 21). All group comparisons were statistically significant (*p < 0.05). Panels **b**, **c** show scatter plots that relate the HBSI with two cardiovascular autonomic parameters, deep breathing E/I ratio (left) and Valsalva PRT (right), (**b**), and with autonomic manifestations (total SCOPA-AUT) (**c**). The statistical significance and values of r (Rs) of the correlations calculated independently for patients and controls are shown in each graph, as well as the corresponding adjusted regression lines. All represented variables are dimensionless except for Valsalva PRT, measured in seconds. PD Parkinson’s disease, PD sev-dys PD patients with severe dysautonomia, PD mod-dys PD patients with moderate dysautonomia, PD mild-dys PD patients with mild dysautonomia, Valsalva PRT Valsalva pressure recovery time, Deep breathing E/I ratio expiratory-to-inspiratory ratio for heart rate variability.*p < 0.05; **p < 0.01; ***p < 0.005.

To establish the clinical severity of the cardiovascular dysautonomia in patients who also underwent clinical autonomic tests (*n* = 30), we constructed a cardiovascular dysautonomia severity composite using the deep breathing E/I ratio (which represents cardiovagal control and was significantly lower in patients) and Valsalva PRT and ΔSBP phase IV (representing both sympathetic functions and being significantly higher in patients). Using this composite, 8 of the patients were classified as severe dysautonomia (sev-dys), 14 as moderate dysautonomia (mod-dys), and 8 as mild dysautonomia (mild-dys) (see Methods). Compared to *mild-dys* PD patients, *sev-dys* PD patients were significantly older (*sev-dys*: 64.3 years; *mild-dys*: 48.9 years; *p*: 0.002), had longer disease duration (*sev-dys*: 8.5 years; *mild-dys*: 3.7 years; *p*: 0.005), and greater motor disability (UPDRSIII *sev-dys*: 33.9; UPDRSIII *mild-dys*: 18.9; *p*: <0.001), an expected finding as greater age, disease duration and motor disability are known to be associated with more severe cardiovascular autonomic manifestations in PD. In order to define at the single-subject level a heart-brain synchronization measure that could be correlated with the clinical dysautonomia severity composite, we also created a new metric that we called the heart-brain synchronization index (HBSI) (see Methods). Accordingly, we observed an intergroup gradient in such HBSI, being lowest for *sev-dys* PD patients, followed by *mod-dys* PD, then by *mild-d*ys PD, and the highest for controls (Fig. 3a). In fact, all pairwise comparisons between groups of patients with different dysautonomia severity and controls were statistically significant. When looking at the association towards clinical autonomic outcomes, the three variables, Valsalva PRT (r = −0.59, *p* < 0.001), deep breathing E/I ratio (r = 0.66, *p* < 0.001), and SCOPA-AUT (r = −0.60, *p* < 0.001), significantly correlated with the HBSI in the PD group (Fig. 3b, c), but neither of them was significantly correlated in controls.

## Discussion

In the present study we evaluated the abnormalities in the synchronization between brain activity and heart rate in PD by simultaneously acquiring BOLD signals from brain fMRI and HRV metrics from finger photoplethysmography in 31 PD patients and 21 controls. To maximize the consistency and accuracy of analyses, we applied an innovative approach that accounted for Pearson lagged cross-correlations and multiple window lengths of HRV-BOLD data. Moreover, for assessing the clinical impact of such heart-brain synchronization abnormalities, we correlated at the individual subject level the resulting HRV-BOLD synchronization measure (HBSI) with well-established dysautonomia severity outcomes. Our main finding was that PD patients had a HRV-BOLD synchronization breakdown in several brain regions belonging to the CAN or indirectly involved in autonomic modulation. Moreover, the amount of synchronization breakdown, as measured by the HBSI, was significantly correlated in patients, but not in controls, with dysautonomia severity outcomes associated with both sympathetic and cardiovagal (parasympathetic) functions (Valsalva PRT, E/I ratio, and SCOPA-AUT). Altogether, our findings support the existence in PD of a functional disconnection during rest between the chronotropic heart function and the activity of CAN, which is correlated with greater clinical severity of dysautonomia.

Since the first pioneering studies on heart-brain interplay by Claude Bernard in the 19th century, several researchers have contributed to unveil the anatomical and functional basis of heart control by the brain^16^. In 1993 Benarroch first proposed the term Central Autonomic Network (CAN), based on previous studies in animals and humans using electrical stimulation, focal lesions, or tracer techniques^17^. CAN was described as a group of interconnected areas of the telencephalon, diencephalon, and brainstem, including the prefrontal cortex, anterior cingulate, insula, amygdala, hypothalamus, periaqueductal gray matter, parabrachial regions in the tegmentum of pons and ventrolateral, ventromedial, and dorsomedial medulla, which control preganglionic sympathetic and parasympathetic visceromotor outputs. Importantly, Benarroch highlighted the role of specific CAN regions such as the insula, anterior hypothalamus, nucleus of tractus solitarius, or ventral medulla in the genesis of cardiac arrhythmias and sudden death in neurological diseases, suggesting the central role of CAN in the cardiac rhythm control. With functional neuroimaging, it has been possible to delineate in vivo the anatomical correlates of brain activity for HRV changes induced by cognitive, emotional, or physical tasks^22^, or at rest^23–25^. In a model proposed by Thayer^16^, the right mPFC, in an interconnected network with the cingulate cortex and the insula, exerts through GABAergic projections to the central nucleus of the amygdala (CeA) a tonic inhibitory control of subcortical sympathetic cardioacceleratory outputs that the hypothalamus, periaqueductal gray matter, and the parabrachial pontine nuclei have on the rostral ventrolateral medulla, and allows the activation of parasympathetic cardioinhibitory neurons of the medulla in the nucleus ambiguus and the dorsal motor nucleus of vagus. In turn, the inactivation of the right mPFC facilitates the activity of the CeA, which, by reducing the inhibition of the nucleus of the solitary tract and the caudal ventrolateral medulla, favors the sympathetic cardioacceleratory activity of the rostral ventrolateral medulla, and simultaneously inhibits the parasympathetic cardioinhibitory motor nuclei of the medulla. Accordingly, the activation of the right mPFC would be associated with an increased vagal parasympathetic cardioinhibitory activity, while its inhibition would result in an increased sympathetic cardioacceleratory activity.

Cardiovascular autonomic manifestations in PD are thought to be a consequence of the injury of both the peripheral and central autonomic nervous system^26^. There is evidence that HRV abnormalities in PD occur early in the course of the disease. In fact, a population-based study found that decreased HRV was a risk factor for the development of PD^14^ and it was shown that patients with idiopathic REM sleep behavior disorder, considered a prodromal phase of PD, have abnormal HRV^8^. However, while myocardial scintigraphy studies support the existence of sympathetic myocardial denervation from premotor phases of PD^31^, its influence on cardiac function and HRV abnormalities is not fully understood^27^. Autopsy studies in PD show accumulation of Lewy bodies and neuronal loss in brain structures from CAN, including regions related to HRV control, such as the insula, anterior cingulate, amygdala, hypothalamus, and brainstem nuclei, mainly the dorsal motor nucleus of vagus^26^. Regarding mPFC involvement in PD, although Lewy body pathology does not occur in the prefrontal cortex until the late stages^32^, neuroimaging studies support an early mPFC dysfunction in PD^33^. This functional mPFC alteration has been linked with early cognitive and affective symptoms in PD and is thought to be a consequence of an abnormal modulation of dopaminergic, cholinergic, serotoninergic, and noradrenergic projections from the pars compacta-substantia nigra, Meynert’s nucleus basalis, raphe nuclei, and locus coeruleus, respectively^34^. Importantly, these brainstem and basal forebrain nuclei are vulnerable and intensely affected by Lewy body pathology in early PD stages^35,36^. Given the leading role in HRV control of mPFC, it could be speculated that the mPFC dysfunction associated with the early neurodegeneration of brainstem/basal forebrain nuclei could be a key mechanism explaining HRV abnormalities in premotor PD.

Despite such evidence supporting CAN involvement in PD, few studies have evaluated in vivo with neuroimaging the relationship between brain integrity and autonomic manifestations in PD^24,37,38^. More specifically, only two studies investigated the relationship between HRV and brain activity in PD. W. Lin et al.^37^ evaluated the relationship between brain perfusion at rest and cardiovascular autonomic tests obtained outside the MRI machine, observing that PD hypo-perfusion of the mPFC and insula significantly correlated with heart rate response to deep breathing. In line with this, we also observed that the deep breathing E/I ratio measured outside the MRI machine was one of the autonomic variables that best correlated with the HBSI. Tessa et al.^24^ simultaneously recorded BOLD signals from brain rs-fMRI and HRV from PPG. Specifically, they performed voxel-wise correlations between rs-fMRI time series and the HF component of HRV, associated with cardiovagal activity. Similar to our results, Tessa et al.^24^ identified numerous cortical and subcortical brain regions with lower HRV-BOLD correlations in PD than in controls, including CAN regions related to HRV modulation such as mid and anterior cingulum, insula, and brainstem, and other regions traditionally not considered as part of CAN, being the cerebellum remarkable for coinciding with our results. Actually, the influence of the cerebellum on the autonomic control of blood pressure has been known for years from experimental studies in animals^21^, especially in relation to the response to head movement and vestibular challenges and to the coupling between blood pressure and breathing, and multiple MRI studies in humans now support its role in the sympathetic control of blood pressure related to postural changes^39–41^. In PD the cerebellum is being increasingly recognized as an important structure to explain motor and non-motor symptoms^42^. Although neither neuronal loss nor neurodegeneration have been identified in PD in such location, as is the case in multiple well-known regions belonging to CAN^26^, alpha-synuclein aggregates and Lewy neurites, and to a much lesser extent Lewy bodies^43^, as well as denervation of cerebellar dopamine receptors^44^ have been detected in the cerebellum of PD patients. Moreover, in vivo neuroimaging studies have shown cerebellar atrophy and abnormal functional connectivity (FC) between the cerebellum and sensorimotor, dorsal attention, and default mode networks in PD^45^, with cerebellar FC alterations seen at early-stage drug-naïve PD^46^. Considering such cerebellar abnormalities in PD, the known influence of the cerebellum on the physiological coupling between blood pressure and breathing (a key modulator of HRV^47^), and our findings and those by Tessa et al.^24^, it is plausible to argue that disease-associated cerebellar abnormalities play an important role in the HRV abnormalities seen in PD, even in those observed at early PD stages.

In contrast to the work by Tessa et al.^24^, we also found de-synchronization in the mPFC, in the hypothalamus, and much more extensive involvement of the brainstem. Such differences between both studies may be related to the fact that in our work we evaluated the synchronization between brain and HRV at multiple window lengths over which the different HRV metrics are calculated, thus increasing the sensitivity and generalizability of analyses. Taking into account the short disease duration of our study participants (average: 5.6 years) and the marked involvement of mPFC, brainstem, cerebellum, and insula in *NNiqr*-BOLD sync-diff maps, our results support the hypothesis that altered FC of such structures, especially mPFC, brainstem, and cerebellum, could explain the HRV alterations observed in early phases of PD.

Another differential aspect of our work is that we assessed the association between heart-brain de-synchronization maps and clinical outcomes for dysautonomia severity, including objective cardiovagal (deep breathing E/I ratio) and sympathetic (Valsalva PRT) variables, and autonomic manifestations (SCOPA-AUT). The association between the HBSI and Valsalva PRT and deep breathing E/I ratio reinforces the hypothesis that the *NNiqr*-BOLD Sync Maps account not only for the cardiovagal but also the sympathetic HRV control. Given that both sympathetic and parasympathetic manifestations of PD have been associated with a worse prognosis^3^, we believe that our work provides a more representative approach to the central dysautonomia in PD than previously published literature. Importantly, we observed significant differences in HBSI between controls and each of all dysautonomia severity PD subgroups and an inverse exposure–response relationship between dysautonomia severity and HBSI, with HBSI being the lowest for PD with *sev-dys*, followed by PD with *mod-dys*, PD with *mild-dys* and, lastly, controls (Fig. 3). The observed association of the *NNiqr*-BOLD sync-diff maps with Valsalva PRT and deep breathing E/I ratio, two cardiovascular autonomic parameters typically affected in the early stages of PD^48,49^, further supports the idea that HRV-BOLD synchronization analysis could be an early biomarker of dysautonomia in PD. Moreover, among all the clinical autonomic outcomes, the deep breathing E/I ratio was the variable that had a stronger correlation with HBSI and that, when related to HBSI, better separated the PD subgroups of dysautonomia severity. For this reason, we believe that the combination of deep breathing E/I ratio and HRV-BOLD synchronization analysis could be a powerful complementary early marker of central autonomic dysfunction in patients with PD and other neurodegenerative diseases of the CNS presenting dysautonomia, such as pure autonomic failure (PAF) or multiple system atrophy (MSA).

Our work has some limitations. First, we did not analyze the structural or microstructural brain integrity with MRI, which hampered the evaluation of the potential relationship between the dysfunction and the structural damage of the CNS. In addition, we cannot confirm whether the observed alterations in patients were present in very early or premotor phases or whether the origin of the heart-brain synchronization disruption was exclusively related to CAN dysfunction and/or degeneration. Another limitation is a possible underestimation of the HRV metrics due to the short duration of PPG recordings, which lasted the same as the rs-fMRI sequence (7.40 min). This bias was more important for HRV metrics related to sympathetic modulation such as *LF*, which require longer recording times than those metrics subjected to cardiovagal control such as HF^50^. Lastly, although significant correlations were found in PD patients between HBSI and a set of clinically relevant autonomic outcomes, the fact that rs-fMRI and such autonomic outcomes were neither acquired simultaneously nor on the same day, forces us to clarify that such association should be interpreted with caution.

In summary, our findings support the existence of a functional disconnection at rest between the chronotropic function of the heart and the activity of CAN in the brain of PD patients, which is associated with the clinical severity of dysautonomia. Such abnormality in heart-brain synchronization, detected by the simultaneous and synchronous acquisition of rs-fMRI and PPG data, might become a complementary biomarker for early detection of central autonomic dysfunction in PD and in other CNS neurodegenerative conditions such PAF or MSA. Further studies including patients in early or prodromal phases of PD, PAF, or MSA and with multimodal brain and heart imaging are required to support the potential interest of heart-brain synchronization studies in parkinsonism and related disorders.

## Methods

### Participants, study design, demographics, and PD-related features

We performed a cross-sectional study of 31 patients with idiopathic PD and 21 age-matched healthy controls. Participants were recruited in the Department of Neurology at Cruces University Hospital (Basque Country, Spain). Patients fulfilled the UK Parkinson’s Disease Society Brain Bank criteria for the diagnosis of PD^51^. The controls were spouses or companions of the patients. Participants with diabetes mellitus, heart diseases potentially influencing hemodynamic measures, or any other neurological disorder were excluded. Patients were studied in an on-medication condition. Two neurologists experienced in the field of movement disorders recorded patients’ age at disease onset, disease duration, scores of the Hoehn and Yahr scale (HY) and Unified Parkinson’s Disease Rating Scale (UPDRS I–IV) and Levodopa equivalent daily dose (LEDD). General cognitive status was assessed using the MoCA. The study protocol was approved by the regional clinical research ethics committee. All participants gave written informed consent prior to their participation in the study, in accordance with the tenets of the Declaration of Helsinki as revised in 2013.

### Autonomic manifestations and cardiovascular autonomic function tests

Thirty patients and thirteen controls completed an additional visit within less than 1 month from MRI acquisition. This visit consisted of an assessment of autonomic manifestations, including SCOPA-AUT^52^ and non-invasive measurement of heart rate and blood pressure changes in response to standard autonomic tasks (deep breathing, Valsalva maneuver and tilt table) using a Task Force Monitor (CNSystems, Graz, Austria). The SCOPA-AUT scale is self-completed by patients and consists of 25 items with a total score ranging from 0 to 69 points and assessing six domains of autonomic function: gastrointestinal (7), urinary (6), cardiovascular (3), thermoregulatory (4), pupillomotor (1), and sexual (2 items for men and 2 items for women)^53^. We used the SCOPA-AUT total score as a measure of the global integrity of CAN. Autonomic tasks were performed following standard procedures for quantitative autonomic testing^54^. In brief, heart rate and blood pressure variability were continuously monitored at rest in a supine position for 10 min and in response to deep breathing, the Valsalva maneuver and a 20 min tilt test. Four-lead electrocardiography was used to measure the heart rate and inter-beat interval. The E/I ratio was calculated using results from the deep breathing test, which consisted of a series of six successive deep breath cycles in supine position. This was followed by the Valsalva maneuver performed at an expiratory pressure of 40 mmHg for 15 s. We only considered hemodynamic parameters from phase II, late phase II, and phase IV of the Valsalva maneuver, allowing us to calculate Valsalva PRT, which is the recovery time of systolic blood pressure (SBP) from the bottom of phase III to baseline, and Valsalva ratio, computed as the duration of the longest R–R interval in phase IV divided by the duration of the shortest R–R interval between phase II and the very beginning of phase III. After the Valsalva maneuver, patients were instructed to breathe at a normal and comfortable rate. Finally, the 20 min tilt test was performed on a table tilted at an angle of 60°. Orthostatic hypotension was defined as a drop in SBP ≥ 20 mmHg and/ or a reduction in diastolic blood pressure (DBP) ≥ 10 mmHg after 5 min of inclination, according to the criteria from the Consensus Committee of the American Autonomic Society and the American Academy of Neurology^55^. Accordingly, the following autonomic testing parameters were selected for exploring their correlation with the HRV-BOLD synchronization: Valsalva PRT, Valsalva ratio, the change in Valsalva of blood pressure from baseline to phase II late (ΔSBP phase II and ΔDBP phase II) and phase IV (ΔSBP phase IV), deep breathing E/I ratio and the presence of OH. Both the deep breathing E/I ratio and the Valsalva ratio account for the cardiovagal modulation of heart rate during breathing, while ΔSBP and ΔDBP of phase II late, Valsalva PRT and ΔSBP phase IV are associated with the sympathetic vasopressor effects on blood pressure during Valsalva^54,56^.

### HRV-BOLD acquisition, preprocessing, lagged cross-correlations, and Sync Maps

Simultaneous and synchronous BOLD and pulse pressure time series data were acquired and processed as schematically illustrated in Fig. 1.

First, pulse pressure and BOLD signals were concurrently recorded under resting situation during a 7.40 min session, respectively with MRI-compatible finger PPG and with a 3-Tesla brain fMRI. More specifically, brain MRI was acquired in a Philips 3-Tesla Achieva Dstream MRI scanner with a 32-channel head coil. The resting-state fMRI sequence had the following characteristics: during a 7.40 min session and using SENSitivity Encoding (with a factor of 2.2), 214 whole-brain gradient echo-planar images were acquired, each one including 45 axial slices, interleaved in ascending order and with TR/TE = 2100/27 ms, FOV = 240 × 240 mm^2^, voxel size = 3 × 3 × 3 mm^3^, 80 × 80 matrix and slice thickness of 3 mm. In addition, immediately before the rs-fMRI sequence, High resolution T1 images with a 3D Turbo Field Echo (TFE) were acquired with the following parameters: 300 contiguous sagittal slices covering the entire brain and brainstem with repetition time (TR) = 7.4 ms, echo time (TE) = 3.4 ms, inversion time (IT) = 850 ms, voxel size = 1.1 × 1.1 × 1.2 mm^3^, field of view (FOV) = 250 × 250 mm^2^. Simultaneous and synchronously with the rs-fMRI image acquisition, pulse pressure signal was continuously co-recorded at a sampling frequency of 500 Hz on the index finger of the participants’ right hand using a built-in Philips MRI-compatible PPG system, ensuring heartbeats were visible in its signal. BOLD signal from rs-MRI yielded 214 timepoints with 2.1 s as TR, which is equal to 449.4 s (7 min and 29.4 s) per voxel, while PPG signal yielded 224,700 timepoints with 500 Hz as sampling rate, which is equal to 449.4 s (7 min and 29.4 s).

Next, PPG and BOLD signals were separately preprocessed by specific procedures detailed in the following lines. PPG signal was preprocessed with Physionet Cardiovascular Signal Toolbox (PCST) and with MATLAB R2019a. PCST is an open-source modular program for calculating HRV metrics and PPG preprocessing^57^. For the global settings of PCST, we chose a varying window length with values corresponding to different integer multiples of the repetition time (TR) (2.1 s) of the BOLD signal. The number of time series segments with lowest HR was set to 5. The percentage of data that can be rejected before a window is considered too low quality for the analysis was set to 35%, and the same amount was also fixed for the maximum percentage of data allowable to be missing from a window. In the case of the settings for the SQI (Signal Quality Index established in the Toolbox), the Low-Quality Threshold was set to 0.8, and the window length and the increment were set as for the global settings. Time threshold was set to 0.1 s and the margin time to 2 s. For peak detection, the refractory period between two R-peaks was set to 0.25, the energy threshold of the detector to 0.6, and the size of the windows to perform the QRS detection to 15 s (looking at positive signs of the peaks). Since pulse variability has been shown to provide a good estimator of HRV, especially to study autonomic function in resting situations^58^, the present work assesses the pulse variability signal to calculate all different metrics from HRV. Therefore, throughout the manuscript we referred to pulse rate variability as HRV. In line with the usual terminology for continuous ECG recordings analysis, the normal beat-to-beat intervals of the pulse variability signal were also termed as normal-to-normal (NN) intervals. The identification of motion artifacts, detection errors or ectopic beats in PPG signals was carried out in different steps. PPG signal outliers, drifts, offset and movement artifacts were removed by mean-removal detrending. The signal was further processed to remove the effect of the respiratory rhythm and higher frequency disturbance by applying band-pass filtering at the frequency range of 0.6–15 Hz. After performing noise and motion correction of PPG signals, peaks of PPG waveforms were automatically detected, and SQI was assessed by default functions in PCST. Each of the calculated HRV metrics was computed for a total of ten window lengths (WL) that corresponded to consecutive multiples of TR in the rs-fMRI, i.e., WL of time equal to 2.1, 4.2, 6.3, 8.4, 10.5, 12.6, 14.7, 16.8, 18.9, and 21 s. The processing of PPG signals with the PhysioNet toolkit resulted in a set of vectors for the different HRV metrics, achieved by adjusted timepoints (Adjusted Time-Points, AdjTP) that were calculated as (215–ceiling (WL/2.1)), which corresponded to the starting time-instants for the window analyses performed in time-increments of TR = 2.1 s. Eight different HRV metrics were obtained for our analyses. Three of them are considered as linear time-domain metrics: *SDNN* (that is, all intervals between adjacent QRS complexes resulting from sinus node depolarizations), *RMSSD* and its *NNiqr*. *SDNN* and *RMSSD* are two of the most widely investigated time-domain HRV measures^59^. *SDNN* is thought to represent the balance of sympathetic and cardiovagal modulation of HRV, while *RMSSD* is intimately associated to respiratory modulation of HRV and respiratory sinus arrhythmia (RSA), which are strongly driven by tonic cardiac vagal (parasympathetic) activity^28^. Although the physiological meaning of *NNiqr* regarding autonomic control of HRV is less documented, it is expected to have a similar interpretability as *SDNN* since the interquartile range is also a descriptive measure of dispersion in the data. Two of the eight final metrics were frequency-domain: the *HF* (Fr: 0.15 Hz ≤ *HF* < 0.4 Hz) and the *LF* (Fr: 0.04 Hz ≤ *LF* < 0.15 Hz). *HF* has been consistently associated to RSA, mostly due to vagal activity in resting conditions, while *LF* has been related to baroreflex activity, controlled by both sympathetic and parasympathetic systems^28^. The last three of the eight metrics were considered as non-linear, obtained from recurrence plots, which are phase-space representations of the values of the NN time series with respect to either the backward or forward time-shifted NN time series. Here, we represented NN values at time *t* as a function of the preceding ones at time *t-1*, a.k.a. Poincare plot. After fitting an ellipse to the represented points, we calculated the dispersion of the data in the orthogonal direction to the identity (*y* = *x*) (the minor axis of the ellipse, *SD1*), the dispersion of the data in the same direction as the identity (the major axis of the ellipse, *SD2*), and the multiplication of the two, *SD*1 × *SD*2, a proxy of the area of the ellipse. *SD1* assesses short-term HRV and correlates with baroreflex sensitivity (BRS), which is the change in NN interval duration per unit change in blood pressure, and with *HF* power; *SD2* measures short- and long-term HRV and correlates with *LF* power and BRS; *SD*1 × *SD*2 is interpreted as a measure of the total HRV, equally representing *SD1* and *SD2* changes^12,29,30^. When assessing group differences in HRV-BOLD synchronization maps, *NNiqr* provided statistically significant differences across window lengths, being the only HRV metric that achieved such a performance as compared to the others, indicating robustness across scales of its heart-brain de-synchronization patterns. When looking to the relation between *NNiqr* and other metrics (Supplementary Fig. 1), we found significant correlations with HF (average across windows r: +0.76), SD2 (r: +0.73), SD1 × SD2 (r: +0.72), and RMSSD (r: +0.69). Accordingly, because it has been shown that HF and RRSSD account for cardiovagal HRV modulation, and SD2 and SD1 × SD2 for both sympathetic and cardiovagal HRV control, and since *NNiqr* is significantly correlated with such all those HRV parameters, it is reasonable to argue that *NNiqr* is a parameter with mixed cardiovagal and sympathetic correspondence on HRV modulation. *NNiqr* is a descriptive measure of dispersion of data, which also accounts for deviation from Gaussian variability of signal. Moreover, from a statistical point of view, the advantage of interquartile range over other data variability metrics is that the former is more robust for skewed or not normalized data and therefore is less affected by outliers^60^, an advantage highlighted as a convenience for HRV studies^61^. The rs-fMRI images were preprocessed with FSL 6.0.1^62^ and AFNI 19.3.00 (https://afni.nimh.nih.gov/) as previously described^63^. In brief, slice-time correction was applied, and volumes were aligned for head motion artifact correction with MCFLIRT (FSL). Movement time-courses obtained from MCFLIRT were used as an input of the ICA-AROMA^64^, a data-driven method used to identify motion-related independent components (IC) from fMRI data. After intensity normalization, time-courses of the motion-related ICs, average cerebrospinal fluid and white matter signals, and linear and quadratic trends were removed together with a 0.01–0.08 Hz band-pass. Finally, images were normalized to the MNI152 brain template (3 × 3 × 3 mm^3^ voxel size) and spatially smoothed with a 6 mm full width at half maximum isotropic Gaussian kernel.

Before calculating the amount of synchronicity between BOLD and HRV metrics, we down-sampled the HRV signal (acquired at 500 Hz) to the same frequency as the one for fMRI (1/TR = 0.47 Hz). Next, we calculated the different HRV metrics per window, using a sliding 80%-overlapping window analysis. Window lengths (WL) were varying, and equal to multiples of the TR used in the fMRI (see Methods). As a result, the HRV metric time series had equal length to the BOLD signals. Next, for each WL, eight different HRV metrics (*SDNN*, *RMSSD*, *NNiqr*, *HF*, *LF*, *SD1*, *SD2,* and *SD*1 × *SD*2) were used to create one HRV-BOLD synchronization map per participant, representing the voxel-wise maximum lagged cross-correlations between the BOLD signal and the time series of each HRV metric. Lagging was used to control for the regional variability in the physiological delay between the two classes of time series, which is, a priori, unknown. The HRV-BOLD lagged cross-correlation 3D brain maps were referred as HRV-BOLD Sync Maps that, after smoothing them with a Gaussian spatial filter of sigma equal to 3 (equivalent to FWHM = 7 mm), were finally used for group comparison.

### Group comparison of HRV-BOLD Sync Maps

A General Lineal Model was fitted for each voxel of the 3D HRV-BOLD Sync Map (one per HRV metric and WL), and group comparisons were performed with two different contrasts: patients > controls and controls > patients. For multiple comparisons, we restricted the analyses to the voxels belonging to a 3 mm binary mask of brain gray matter. Correction for multiple comparisons was performed with a cluster-based thresholding method using *3dClustSim* of AFNI 19.3, which uses a Monte Carlo simulation with 10,000 iterations to estimate the probability of false positive clusters. After group comparison for each of the HRV metrics, the Fisher’s statistics was used to pool together the different _*pi*_ values, each one obtained for a different WL. Thus, assuming *k* different WLs, the individual _*pi*_ values for each voxel were transformed into a chi-squared statistic of *2k* degrees of freedom, following $\chi _{2k}^2 = - 2\mathop {\sum}\limits_{i = 1}^k {\ln p_i}$. Although the goodness of this procedure requires independence in the different observations providing the _*pi*_ values, and this was not generally fulfilled as in our case corresponded to different WL-dependent HRV metrics, our strategy achieved to pool all the group differences WL-maps into a final one, that after Bonferroni-correction, was used for further analyses.

### Brain MRI atlas for Central Autonomic Network

For precise identification of the anatomical relations between the resulting HRV-BOLD Sync Maps and the CAN, we created a custom MRI atlas of the CAN by using a collection of regions extracted from several public MRI atlases. The custom CAN atlas included the following regions: mPFC (built with bilateral Medial orbitofrontal, Superior frontal and Frontalpole labels from Deskian-Killiany atlas)^65^, Anterior Cingulate Cortex (ACC) (built with bilateral rostral anterior cingulate cortex and caudal anterior cingulate cortex labels from Deskian-Killiany atlas), Insula (Deskian-Killiany atlas), Amygdala (Deskian-Killiany atlas), Bed nucleus of stria terminalis^66^, Hypothalamus^67^, Periaqueductal gray (PAG)^68^, Parabrachial complex (PBC)^68^, and Medulla^69^ (Fig. 2).

### Correlation of clinical autonomic outcomes and heart-brain synchronization index

The HBSI was defined to assess at the single-subject level a measure of the statistical significance of the amount of heart-brain synchronization. To do so, we constructed a null-hypothesis distribution at the voxel-level by performing 10,000 random permutations on time series of the HRV metric and computed for each surrogate time series its maximum lagged Pearson cross-correlation value with each of the BOLD voxel time series. The number of 10,000 permutations ensured stability for the estimation of the *p*-value. For each voxel, we then calculated the significance of the actual max-lagged cross-correlation value (obtained between actual non-surrogate HRV time series and the BOLD voxel-level time series) by calculating one *p*-value within the surrogate distribution. The HBSI was defined simply by counting the number of significant voxels (*p* < 0.05) that existed in a certain region of interest, and normalizing it between all the values of healthy controls and patients. For group comparisons (Fig. 3a) such a region corresponded to that one defined by the contrast patient < control in the HRV-BOLD Sync Maps group comparisons. In contrast, for the association with autonomic clinical outcomes (Fig. 3b), the region of interest was defined as the intersection between the regions set by the contrast patient < control and those established by the association of each clinical outcome and the HRV-BOLD Sync Maps. Next, to understand whether the HBSI was modulated by the degree of dysautonomia in patients, we assessed associations separately in a subgroup of patients with *sev-dys*, with *mod-dys*, with *mild-dys*, and in controls. The strength of dysautonomia was quantified by a cardiovascular dysautonomia severity composite including the clinical parameters of the autonomic tests for which there were statistically significant differences (≤0.05) between patients and controls: deep breathing E/I ratio, Valsalva PRT and ΔSBP phase IV (see Supplementary Table 1). The composite was obtained by combining the respective z-scores as ${{{\mathrm{z}}}}({{{\mathrm{E}}}}/{{{\mathrm{Iratio}}}}) + {{{\mathrm{z}}}}({{{\mathrm{SBPphaseIV}}}}) - {{{\mathrm{z}}}}({{{\mathrm{Valsalva}}}}\,{{{\mathrm{PRT}}}})/3$. This dysautonomia composite allowed the definition of *sev-dys* patients as those with a value in the composite belonging to the 1st-quartile of the distribution of values, *mild-dys* patients as those belonging to the 4th-quartile, and the rest of patients were considered as *mod-dys*. Analyses were repeated separately for such three PD subgroups and controls.

### Reporting summary

Further information on research design is available in the Nature Research Reporting Summary linked to this article.

## Supplementary information

Supplementary information

Reporting Summary Checklist

## Data Availability

The data and the source-code of the analysis that support the findings of this study will be available after publication from the corresponding author, upon reasonable request.
