# Supplementary material for: Heart-brain synchronization breakdown in Parkinson’s disease
Source: NPJ Parkinsons Dis. 2022 May 30;8:64. doi: 10.1038/s41531-022-00323-w (PMC9151654; doi:10.1038/s41531-022-00323-w)
Supplement: Supplementary file 1 — Supplementary information [file 41531_2022_323_MOESM1_ESM.pdf]

Supplementary information

Supplementary Table S1. Demographics, neurological and autonomic outcomes of participants that completed autonomic tests

	Controls	iPD	p-value
n	13	30	NA
SCOPA-AUT total score	5 (1)	12 (2)	0.005[b]
OH in tilt table test (%)	2 (15.4)	21 (70)	0.070
Deep breathing E/I ratio	1.2 (0.1)	1.1 (0.1)	0.095
ΔSBP phase II late, mmHg	-7.0 (6.6)	-14.4 (2.9)	0.277
ΔDBP phase II late, mmHg	11.3 (5.3)	-15.6 (18.5)	0.209
ΔSBP phase IV, mmHg	28.4 (5.5)	8.9 (3.1)	0.029[b]
Valsalva ratio	1.4 (0.1)	1.4 (0.1)	0.550
Valsalva PRT, sg	2.8 (0.6)	4.5 (0.4)	0.009[b,c,d]

p-values were obtained with Student's t-test. Significant results were corrected with Bonferroni correction. The units of measure for each parameter are indicated in the table except for those that are dimensionless (Deep breathing E / I ratio and Valsalva ratio). iPD, idiopathic Parkinson's disease; UPDRS III: motor part of Unified Parkinson's Disease Rating Scale; HY score: Hoehn & Yahr score; IQR: inter-quartile range; MoCA, Montreal Cognitive Assessment; LEDD, L-Dopa equivalent daily dose; SCOPA-AUT Scales for Outcomes in Parkinson's Disease-Autonomic questionnaire; OH: orthostatic hypotension; E/I ratio: expiratory-to-inspiratory ratio for heart rate variability; SBP systolic blood pressure; DBP diastolic blood pressure; ΔSBP phase II late change of SBP from baseline to phase II late of Valsalva, ΔDBP phase II late change of DBP from baseline to phase II late of Valsalva, ΔSBP phase IV change of SBP from baseline to phase IV of Valsalva, ΔDBP phase IV change of DBP from baseline to phase IV of Valsalva, PRT pressure recovery time; sg seconds.

Supplementary Figure S1. Relations between NNiqr and representative HRV parameters for different time windows (TR)

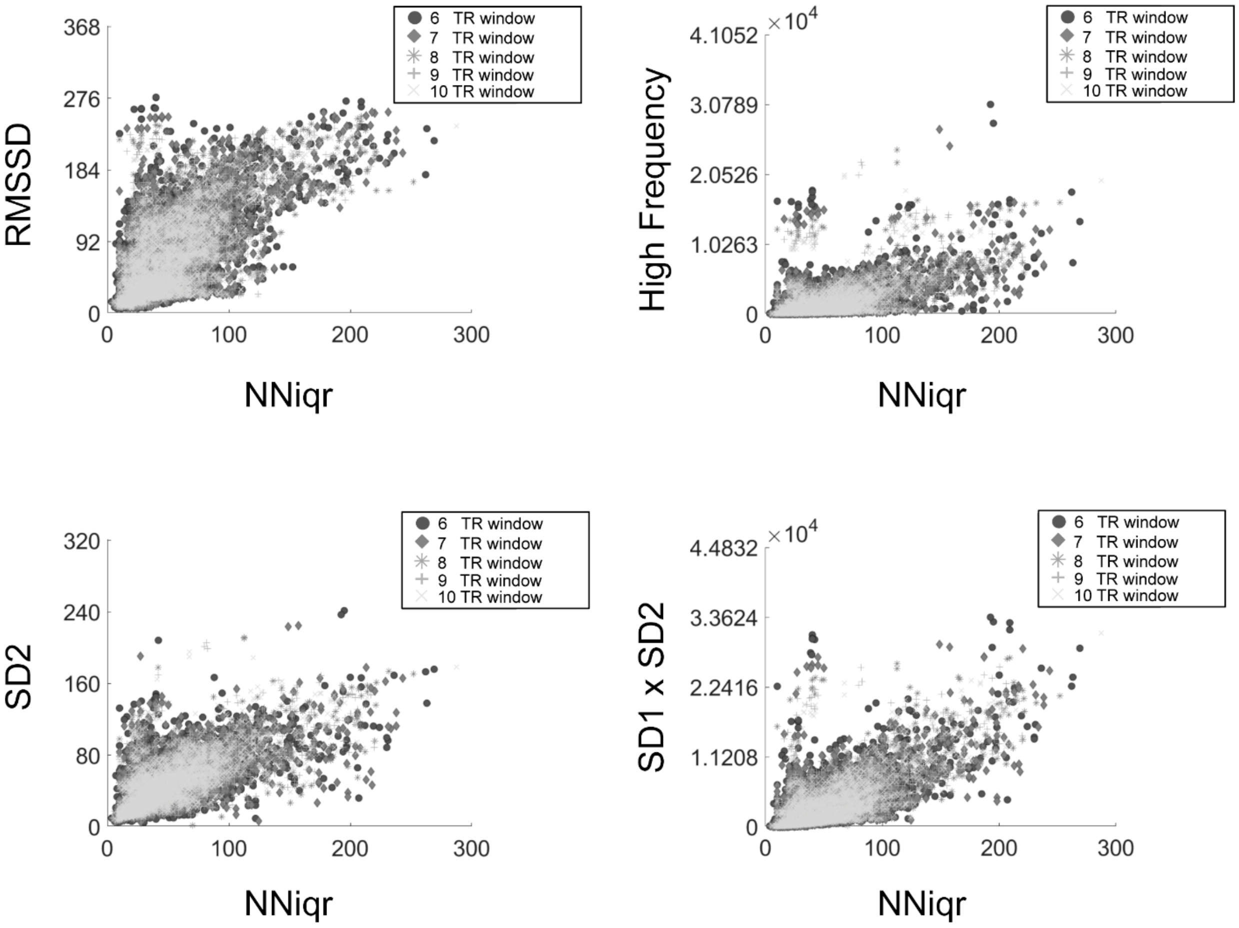

Across different window lengths, NNiqr had significant association with RMSSD, High Frequency (HF), SD2 and SD1xSD2. See the Methods section in the main manuscript for further details.
