# Supplementary material for: Heart-brain synchronization breakdown in Parkinson’s disease
Source: NPJ Parkinsons Dis. 2022 May 30;8:64. doi: 10.1038/s41531-022-00323-w (PMC9151654; doi:10.1038/s41531-022-00323-w)
Supplement: Supplementary file 2 — Reporting Summary Checklist [file 41531_2022_323_MOESM2_ESM.pdf]

nature portfolio

Corresponding author(s): Iñigo Gabilondo

Last updated by author(s): Mar 19, 2022

Reporting Summary

Nature Portfolio wishes to improve the reproducibility of the work that we publish. This form provides structure for consistency and transparency in reporting. For further information on Nature Portfolio policies, see our Editorial Policies and the Editorial Policy Checklist.

Statistics

For all statistical analyses, confirm that the following items are present in the figure legend, table legend, main text, or Methods section.

n/a	Confirmed	
☐	☒	The exact sample size (*n*) for each experimental group/condition, given as a discrete number and unit of measurement
☐	☒	A statement on whether measurements were taken from distinct samples or whether the same sample was measured repeatedly
☐	☒	The statistical test(s) used AND whether they are one- or two-sided<br>*Only common tests should be described solely by name; describe more complex techniques in the Methods section.*
☐	☒	A description of all covariates tested
☐	☒	A description of any assumptions or corrections, such as tests of normality and adjustment for multiple comparisons
☐	☒	A full description of the statistical parameters including central tendency (e.g. means) or other basic estimates (e.g. regression coefficient) AND variation (e.g. standard deviation) or associated estimates of uncertainty (e.g. confidence intervals)
☐	☒	For null hypothesis testing, the test statistic (e.g. *F*, *t*, *r*) with confidence intervals, effect sizes, degrees of freedom and *P* value noted<br>*Give P values as exact values whenever suitable.*
☐	☒	For Bayesian analysis, information on the choice of priors and Markov chain Monte Carlo settings
☐	☒	For hierarchical and complex designs, identification of the appropriate level for tests and full reporting of outcomes
☐	☒	Estimates of effect sizes (e.g. Cohen's *d*, Pearson's *r*), indicating how they were calculated

Our web collection on statistics for biologists contains articles on many of the points above.

Software and code

Policy information about availability of computer code

Data collection	Brain MRI and PPG: Philips 3-Tesla Achieva Dstream MRI scanner with a 32-channel head coil, a built-in Philips MRI compatible photoplethysmography. Non-invasive measurement of heart rate and blood pressure changes in response to standard autonomic tasks: Task Force Monitor (CNSystems, Graz, Austria).
Data analysis	Brain MRI BOLD signal preprocessing: FSL 6.0.1 4 and AFNI 19.3.00<br>PPG signal preprocessing and computation of HRV metrics: Physionet Cardiovascular Signal Toolbox (PCST) and MATLAB R2019a

For manuscripts utilizing custom algorithms or software that are central to the research but not yet described in published literature, software must be made available to editors and reviewers. We strongly encourage code deposition in a community repository (e.g. GitHub). See the Nature Portfolio guidelines for submitting code & software for further information.

Data

Policy information about availability of data

All manuscripts must include a data availability statement. This statement should provide the following information, where applicable:

- Accession codes, unique identifiers, or web links for publicly available datasets
- A description of any restrictions on data availability
- For clinical datasets or third party data, please ensure that the statement adheres to our policy

The data and the source-code of the analysis that support the findings of this study will be available after publication from the corresponding author, upon reasonable request.

Field-specific reporting

Please select the one below that is the best fit for your research. If you are not sure, read the appropriate sections before making your selection.

☒ Life sciences ☐ Behavioural & social sciences ☐ Ecological, evolutionary & environmental sciences

For a reference copy of the document with all sections, see nature.com/documents/nr-reporting-summary-flat.pdf

Life sciences study design

All studies must disclose on these points even when the disclosure is negative.

Sample size	No sample size was calculated.
Data exclusions	Participants with major artifacts on MRI or ppg signal, which could not be reliably corrected, were excluded.
Replication	Until now, we have not had the possibility to replicate the analyzes in a different sample since we have not had access to other comparable PD datasets (that have the simultaneous acquisition of MRI and pulse oximetry, and with autonomic tests)
Randomization	N/A
Blinding	N/A

Reporting for specific materials, systems and methods

We require information from authors about some types of materials, experimental systems and methods used in many studies. Here, indicate whether each material, system or method listed is relevant to your study. If you are not sure if a list item applies to your research, read the appropriate section before selecting a response.

Materials & experimental systems

n/a	Involved in the study
☒	☐ Antibodies
☒	☐ Eukaryotic cell lines
☒	☐ Palaeontology and archaeology
☒	☐ Animals and other organisms
☐	☒ Human research participants
☐	☒ Clinical data
☒	☐ Dual use research of concern

Methods

n/a	Involved in the study
☒	☐ ChIP-seq
☒	☐ Flow cytometry
☒	☐ MRI-based neuroimaging

Human research participants

Policy information about studies involving human research participants

Population characteristics	Patients: n=31, idiopathic Parkinson's disease, mean age 59 years, 11% females, 5.6 years of disease duration, mean UPDRS III 27.3 and MoCA: 24.3. Controls: n=21, age 54.6 years, 9% females, MoCA 26.8
Recruitment	Participants were recruited In the Department of Neurology at Cruces University Hospital (Basque Country, Spain). They were selected during routine clinical visits in consultation based on the inclusion and exclusion criteria of the study, in the period from 2015 to 2017. The controls were relatives or spouses of the patients.
Ethics oversight	Regional (Basque Country) Ethics Commitee: Comité de Ética de la Investigación con medicamentos de Euskadi. https://www.euskadi.eus/comite-etico-investigacion-con-medicamentos/

Note that full information on the approval of the study protocol must also be provided in the manuscript.

Clinical data

Policy information about clinical studies

All manuscripts should comply with the ICMJE guidelines for publication of clinical research and a completed CONSORT checklist must be included with all submissions.

Clinical trial registration	Not registered in Clinicaltrials.gov
Study protocol	This is a non-experimental observational clinical study that is part of a neuroimaging project in Parkinson's. It was partially funded by the Carlos III Health Institute and the Michael J Fox Foundation (MJFF). The specific protocols were sent to these funding entities and to the Euskado Ethics Committee, they were not published on any website. The general project summary can be found on the MJFF

	website: https://www.michaeljfox.org/grant/neuroimaging-carriers-alpha-synuclein-e46k-mutation-model-idiopathic-lewy-body-disease
Data collection	Participants were recruited in the Department of Neurology at Cruces University Hospital (Basque Country, Spain), where the clinical study of the autonomic nervous system was completed. MRI images were obtained in Osatek-Galdakao Hospital (Basque Country, Spain).
Outcomes	The main measurements were the BOLD signal of the fMRI, the HRV parameters obtained from the pulse oximetry signal, the variables of the autonomic tests (deep breathing, head-up tilt and Valsalva) and the SCOPA-AUT questionnaire.
